# Predictors of Drug-Resistant Tuberculosis among High-Risk Population Diagnosed under National Program Conditions in the Littoral Region, Cameroon

**DOI:** 10.1155/2021/8817442

**Published:** 2021-11-19

**Authors:** Teyim Pride Mbuh, Adeline Wandji, Liliane Keugni, Sandrine Mboh, Irene Ane-Anyangwe, Wilfred Fon Mbacham, Henry Dilonga Meriki

**Affiliations:** ^1^Department of Microbiology and Parasitology, Faculty of Science, University of Buea, Cameroon; ^2^Tuberculosis Reference Laboratory Douala, Cameroon; ^3^Littoral Regional Technical Group for the Control of Tuberculosis/Littoral Regional Delegation for Public Health, Cameroon; ^4^Biotechnology Centre of Nkolbisson, University of Yaounde I, Cameroon

## Abstract

Multiple drug resistance TB (MDR-TB) has greatly jeopardized the effective control of tuberculosis in Africa. This study is aimed at determining the incidence and predictors of drug resistant-TB amongst bacteriologically diagnosed cases in the Littoral region of Cameroon. This was a descriptive cross-sectional survey conducted from January 2016 to December 2017. A total of 1665 participants were enrolled from 32 diagnostic and treatment centers (DTCs) in the Littoral region. Demographic, clinical, socioeconomic, and behavioral data were obtained using a pretested structured questionnaire. Drug susceptibility testing was performed using Gene Xpert MTB/RIF assay and line probe assay (LPA). Consent was obtained from participant/guidance. Data analysis was carried with SPSS version 21. Univariate and multiple logistic regression was performed at 5% significance level. The incidence of rifampicin and MDR-TB was 86 (5.2%) and 75 (4.5%), respectively. More (11.3%) cases of drug resistance were diagnosed in 2016 compared to 2017 (3.7%). Eleven (0.7%) were resistant to rifampicin only. A total of 19 (4.4%) cases of rifampicin resistance were detected from newly diagnosed cases and 67 (5.4%) from previously retreated cases. Pre-XDR-TB was detected in 2 (2.7%) of the MDR-TB cases amongst whom 1 (1.3%) was extensive drug resistance TB (XDR-TB). Age greater than 60 years old (OR = 4.98, *p* = 0.047), being married (OR = 1.91, *p* = 0.006), being currently incarcerated (OR = 1.74, *p* = 0.027), and having contact with known TB cases (OR = 1.88, *p* = 0.007) were associated to MDR-TB in a univariate analysis. This study highlights the declining rates of TB drug resistance in the region over the years probably due to the introduction of Gene Xpert that results in early detection of RR-TB. It also shows that age greater than 60 years, being married, and incarcerated are predictors of drug resistant-TB, while the year of patient enrolment and previous exposure to TB treatment were independent predictors of drug resistance in the Littoral region of Cameroon.

## 1. Introduction

Tuberculosis (TB) is a communicable disease caused by *Mycobacterium tuberculosis* complex and transmitted principally by the respiratory route. Globally, persons with an immune compromised HIV/AIDS status are more likely (17-23 times) to develop TB in a lifetime than those who are immune competent [1]. Drug-resistant tuberculosis is a global health challenge that threatened the progress of tuberculosis eradication programs in the world and particularly in Africa. The World Health Organization (WHO) defines MDR-TB as strains of *Mycobacterium tuberculosis* that are resistant to isoniazid and rifampicin, with or without resistance to other first-line drugs [2], and factors, such as the usage of immunosuppressive drug, HIV/AIDS, smoking, alcohol usage, diabetes mellitus, malnutrition, young age, overcrowding, poor housing conditions, and economic deprivation have been associated as predictors of drug-resistant TB [1]. Drug resistance surveillance data show that 3.9% of new and 21% of previously treated TB cases were estimated to have had MDR/RR-TB in 2015 [3]. There was some progress made in testing and detection of MDR/RR-TB cases in the year 2018, literature review shows that globally in 2018, 51% of persons with bacteriologically confirmed TB were tested for rifampicin resistance, and as much as 41% in 2017 [4]. Testing coverage of 46% for new and 83% for previously treated TB patients was recorded, and a global total of 186 772 cases of MDR/RR-TB were detected and notified in 2018 [4].

Despite this progress made, the number of persons on TB treatment in 2018 was equivalent to only one-third of those who developed MDR/RR-TB in 2018. Amongst the many possible ways of closing this gap is the need for an increase in the coverage of testing for drug resistance among bacteriologically confirmed cases, accurate and early detection of drug-resistant tuberculosis is essential for effective patient care, for preventing the development of drug-resistant strains of tuberculosis. Culture-based drug susceptibility tests are the recommended methods for the detection of drug-resistant tuberculosis, but they are time-consuming and technically challenging, especially in resource-limited settings [5] Many nucleic acid amplification assays that detect mutations associated with anti-TB drug resistance have been developed recently, to provide affordable, accurate, simple, and rapid diagnostic tests for DR-TB detection. Cameroon was one of the leading African countries that introduce nucleic acid amplification-based assays in the National Tuberculosis Program, and the Gene Xpert MTB/RIF and Line Probe assays were introduced in the Littoral region in 2014.

According to the WHO Global Report on antituberculosis drug resistance in the World for years [6, 7], MDR-TB strains have emerged in all regions of the world. However, despite, the African continent accounting for over a quarter of new TB cases among people living with TB, MDR-TB appears nearly absent from this continent, which, until recently, reported the lowest median levels of drug resistance TB [8]. Two explanations can be put forward to explain these reported low levels of MDR-TB in the African Continent. The first is the lack of data on drug-resistant TB in most African Countries [8], and the second is the recent introduction of rifampin in Africa. It is often stated that because rifampin was only recently introduced in Africa on a large scale, there has been relatively little time for resistance to develop. However, the development of rifampin resistance may be spurred by HIV infection, and such resistance appears to develop rapidly [8].

This study was therefore conducted to determine the incidence of MDR-TB and XDR-TB and also to predict indicators of drug resistance-TB amongst patients seeking medical attention in the Littoral Region of Cameroon.

## 2. Methods

### 2.1. Study Design and Population

We used a descriptive cross-sectional hospital-based epidemiological survey to screen 3492 participants who were either strong clinical suspects for TB having a smear negative result at the peripheral laboratories or presenting with symptoms of TB having a pre-TB treatment exposure from January 2016 to December 2017. One hundred and ninety (190) were rejected because of incomplete questionnaires or poor specimen quality. A total of 3302 were enrolled. The Littoral region was chosen because it harbors about 20% of all TB diagnosed cases in Cameroon and also because it is the economic headquarter of Cameroon and Douala; the regional headquarter is the largest city in Cameroon with a population of 3,663,000 people [9]. It harbors many referral diagnostic and treatment institutions that pull many persons from the regions in search of better health services.

### 2.2. Laboratory Variables

A total of 3302 (3057 sputum specimens and 245 nonsputum) specimens (sputum, pleural fluid, cerebrospinal fluid, synovial fluid, ascites, and lymph node aspirate) obtained from participants for this study from 32 (82.1%) of the 39 DTCs. They were decontaminated using 3% NaOH, and the pellet used for TB diagnosis by liquid culture (Becton, Dickson and Company, USA, BACTEC 960) for 42 days, LJ slant, was incubated (MEMMERT GmbH, INE 800, Germany) at 37°C for eight consecutive weeks and by Gene Xpert MTB/RIF (Cepheid, France).

The Gene Xpert system integrates and automates sample processing, nucleic acid amplification, and detection of the target sequences. The primers in the Gene Xpert MTB/RIF assay amplify a portion of the *rpoB* gene containing the 81 base pair “core” region. The probes can differentiate between the conserved wild-type sequence and mutations in the core region that is associated with rifampicin resistance (RR). All procedures were performed following manufacturer instructions.

A total 3492 participants were recruited for this study, and 1665 were analyzed for initial drug susceptibility testing (DST) which was rifampicin by Gene Xpert MTB/RIF. Rifampicin resistance cases were further subjected to line probe assay (LPA), Hain Lifescience, Nehren, Germany GenoType MTBDRplus and GenoType MTBDRsl assays (Applied Biosystems thermocycler, life technologies, 2012, Singapore), and DST testing for isoniazid, rifampicin, fluoroquinolones, and injectable aminoglycosides (kanamycin, amikancin, and capromycin).

Following the national policy on HIV testing, all persons with signs and symptoms of TB were offered an HIV test. This was performed following standard national protocols. Briefly, 0.5 ml of plasma was transferred to the absorbent pad of the Determine HIV 1/2 test strip. Positive samples were confirmed on rapid SD Bioline HIV 1/2 3.0 cassettes.

### 2.3. Outcome Variables

Rifampicin resistant-TB case was defined as a TB case diagnosed with resistance to rifampicin, MDR-TB case was a TB case diagnosed with resistance to at least isoniazid and rifampin, pre-XDR-TB was defined as MDR-TB with resistance to either a fluoroquinolone or a second-line injectable agent but not both, and XDR-TB was a TB case diagnosed with resistance to isoniazid and rifampin, plus any fluoroquinolone and at least one of three injectable second-line drugs (i.e., amikacin, kanamycin, or capreomycin).

### 2.4. Independent Variables

Data on demographics (sex, age), clinical (fever, cough, weight loss, and night sweat), socioeconomic (marital status, monthly income below 50.000 FCFA), behavioral (alcohol consumption and cigarette smoking), previous contact with TB cases, and incarceration were obtained using structured questionnaires. A new case was any tuberculosis case that had never taken antituberculosis or treated for less than a month; a relapse was any patient having a second episode of TB; a therapeutic failure was a case whose smear remained or became positive again in the 5th month or later during TB treatment. We adapted the scoring system from a study by de Castro et al. (2011) [10] to assess the association between clinical evidence of TB and the final TB outcome. The variables considered were old age (≤59 years), cough (involuntary, spasmodic, and particularly audible expulsion of breathed air caused by foreign bodies in the larynx or irritation of the mucosa in the trachea and bronchi), weight loss (loss of 3 kg in individuals weighing up to 70 kg and loss of 5 kg in those over 70 kg); fever (body temperature above 37.5°C); night sweats (excessive sweating during the night soaking bedclothes and/or bedding), hemoptysis (expectoration of bright red blood); a history of TB (haven been treated with anti-TB drugs for at least two months), and a total score of ≥7 signified strong clinical evidence for TB disease as described on Table 1.

### 2.5. Ethical Statement

Administrative authorizations for this study were obtained from the permanent secretary for the National Tuberculosis Control Program, and ethical clearance was obtained from the University of Douala ethical review board (No: 1255 IEC-Udo/02/2018/T).

### 2.6. Data Analysis

Data analysis was carried out using SPSS 24.0 (Statistical Package for the Social Sciences, Chicago, Illinois). Univariate analysis was performed with chi-square, and all variables were included in the logistic regression model. Statistical significance was set at *p* < 0.05.

## 3. Results

**Figure 1:**
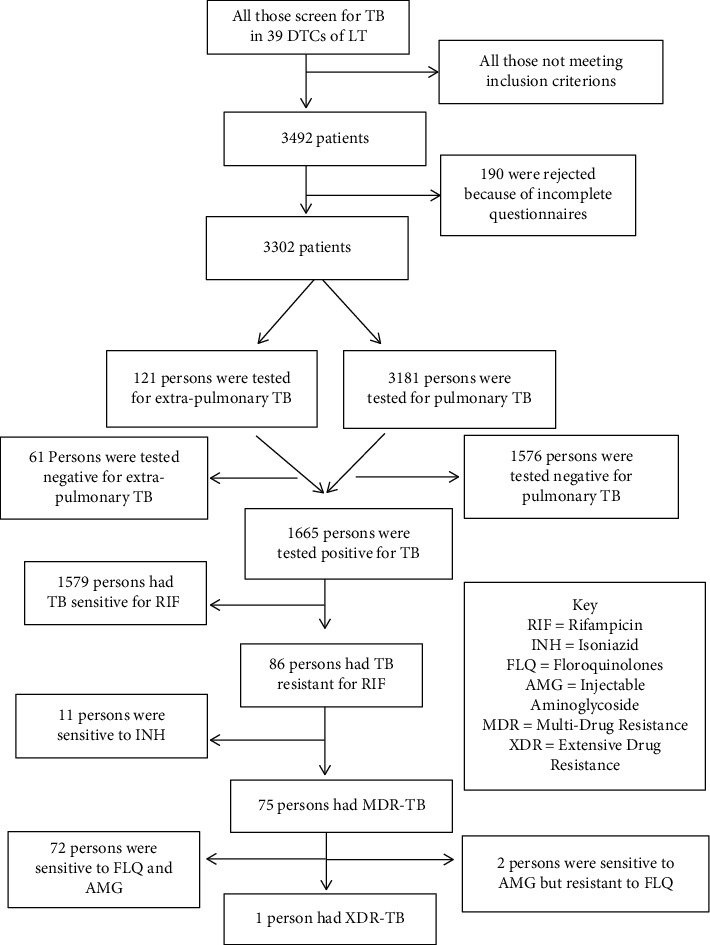
Study profile of participant enrolment and testing.

A total of 3492 participants were enrolled from January 2016 to December 2017 in the Littoral region of Cameroon. A total of 190 (5.6%) participants were not analyzed because of incomplete questionnaires and/or poor sample quality. Overall, 3302 were analyzed among whom 1665 (50.4%) patients were tested positive for *Mycobacterium tuberculosis* as illustrated in Figure 1.

### 3.1. Sociodemographic Characteristics *Mycobacterium tuberculosis-Infected* Participants

The age of participants ranged from 1 to 90 years with a mean age of was 35.85 ± 13.91 years' standard deviation. Most of the participants were male (51.2%) and between the age group 31 and 45 years old (38.4%). Over fifty percent (55.5%) were married, and 17.8% were incarcerated. A total of 71.6% of the participants had been previously infected with tuberculosis of which 63.7% were relapsed cases. The location of the disease was pulmonary (96.4%) in most of the participants, and 26.4% were HIV-positive individuals (Table 2).

### 3.2. Signs and Symptoms of Study Participants at Enrolment

**Figure 2:**
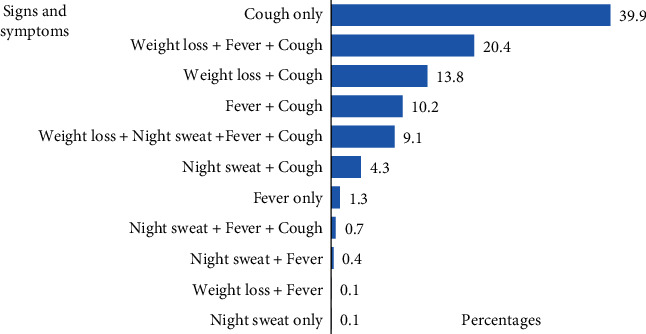
Signs and symptoms in study participants.

Seven specimen types (sputum, bronchoalveolar lavage, cerebrospinal fluid, ascites, lymph node aspirate, pleural, and pericardial fluids) were analyzed in this study with sputum [1543 (92.7%)] being the most frequent followed by bronchoalveolar lavage (3.7%). Cough (98.3%, *n* = 1636) was the most frequent among symptoms presented by participants. A total of 151 (9.1%) patients had all the four cardinal symptoms (cough, weight loss, night sweats, fever) for tuberculosis as illustrated on Figure 2.

### 3.3. Prevalence of RR-TB, MDR, and XDR-TB

**Figure 3:**
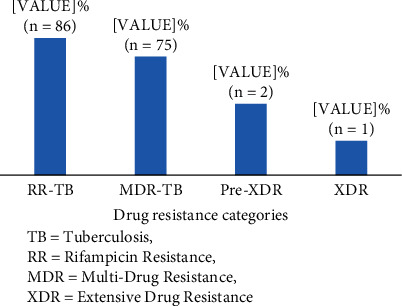
Drug resistance profile of study participants. TB: tuberculosis; RR: rifampicin resistance; MDR: multidrug resistance; XDR: extensive drug resistance.

Of the 1665 patients enrolled, we identified 37 [(11.4%) with previous history for TB and (11.1%) without previous History for TB)] and 49 [(1.7%) with previous history for TB and (4.6%) without previous History for TB)] cases of rifampicin resistance TB (RR-TB) in 2016 and 2017, respectively, making a total of 86 (5.2%) cases of rifampicin resistance. More (11.3%) cases of drug resistance were diagnosed in 2016 compared to 2017 (3.7%). And 11 (0.7%) of them were resistant to rifampicin only. A total of 75 (4.5%) were to both rifampicin and isoniazid (MDR-TB). Of this MDR-TB, 2.7 (2/75) and 1.3% (1/75) were pre-XDR and extensive drug resistance TB (XDR-TB), respectively (Figure 3).

### 3.4. Predictors of Occurrence Drug Resistance TB (All Forms) among Study Participants

The mean age of participants with drug resistance in this study was 38.2 ± 14.3 years, and drug resistance univariate analysis showed that TB drug resistance increased with age with participants older than 60 years having a more than 4 times risk (OR = 4.98, *p* = 0.047) compared to those younger than 16 years. Similarly, married participants recorded more drug resistance TB than unmarried (OR = 1.91, *p* = 0.006) participants. Although not significant (OR = 0.50, *p* = 0.27), drug resistance was predominant among male participants. More drug resistance TB was recorded in the years 2016 than 2017 (11% versus 3.7%, *p* < 0.001). Being incarcerated was significantly associated with developing drug resistance (OR = 1.74, *p* = 0.027). Drug resistance was detected two times more amongst those who had contacts with known TB cases than those who did not have contacts (8% versus 4%, *p* = 0.007). Drug resistance was predominant, though, not significant amongst those with previous TB history (OR = 1.26, *p* = 0.382) as illustrated in Table 3.

## 4. Discussion

Drug-resistant tuberculosis is rising to critically high levels in all parts of the world, and new resistance mechanisms are emerging and spreading globally, threatening our ability to treat the disease. Antibiotic resistance leads to higher medical costs, prolonged hospital stays, and increased mortality. Contemporary data are constantly needed in the monitoring and rational decision-making towards the reduction of TB drug resistance.

This study which mainly targeted suspected patients with TB diagnostic challenges showed that the majority of 1230 (73.9%) of the study participants were preexposured to TB treatment. This was similar to our previous study in the Littoral region of Cameroon [11] but different from that of Meriki et al. [12] in the South West of Cameroon where 88.3% of the study participants were newly diagnosed cases. The prevalence of RR-TB and MDR-TB in this study was 5.2% and 4.8%, respectively. This prevalence is in concordance with global MDR-TB estimates of 2015-2018 [13, 14], however, lower than that report by Jürgen et al. [15] in Cameroon. To the best of my knowledge, Cameroon has not organized a national drug susceptibility testing survey to evaluate drug resistance nationwide. National strategy for drug susceptibility testing in Cameroon from 2009 to 2017 consisted of testing only retreatment cases for drug resistance [16]. The proportion of retreatment cases tested for drug susceptibility moved from 16% in 2009 to 85% in 2017 and the cases diagnosed with MDR-TB moved from 19% in 2009 to 24% in 2011. Since then, it has systematically reduced and was at 13% in 2017 [16]. This data is similar to our findings which demonstrate that RR-TB among retreatment cases reduced from 11.4% in 2016 to 4.6% in 2017. This find could probably be explained by the fact that Gene Xpert was introduced in Cameroon in 2014, and this has led to an improvement in TB case management in Cameroon such that RR-TB cases are diagnosed and treatment early. In this study, RR-TB cases were detected more from TB relapsed (5.7%), failure (4.8%), and newly diagnosed cases (4.4%). Those with pre-TB treatment exposure from whom RR-TB was detected represented 77.9% (67 cases) of all the RR-TB cases. The proportion of RR-TB cases reported in this study is higher than the global average in 2016-2018 [13, 14]. Cameroon being a high HIV burden nation could be an explanation for this observation. In this study, 6.4% of RR-TB cases were coinfected with HIV-1. Studies have shown that the immunocompromised state of HIV patients predisposes them to recurrent TB episodes [17]. HIV patients receiving ART are also at high risk of poor TB treatment adherence due to the high pill burden they take [18]. Some ART drugs have also been documented to have adverse outcomes when administered with anti-TB drugs [18]. These factors combined and increase the risk of failing treatment and developing RR-TB.

Patients with pre-TB treatment exposure were more likely to have RR-TB as compare to new suspects. This finding was in line with that of Ullah [19] who found that the frequency of drug resistance in previously treated TB suspects is 5 times higher than those of newly diagnosed cases. The resistance in new suspects may be indicative of the transmission of resistant strains of the bacilli, while resistance in previously treated cases may be an indicator of poor compliance, lack of treatment supervision, and ineffective TB Control Programme.

Unlike many studies that have demonstrated an association between gender and MDR-TB [20–22], the current study did not show an association between gender and RR-TB. In this study, RR-TB cases were predominantly young with a mean age of 38.2 ± 14.3 years. These findings were similar to those of other studies in Africa [23, 24]. Although a majority of RR-TB patients in the present study belonged to the younger age group (15-45 years), the risk of RR-TB was seen to be almost four times higher among elderly individuals (age>60 years), which concords with the findings of some authors [25, 26] but contrasts with those of others [27, 28] but lower with adjusted odds ratios. This drug resistance data stratified by age is very important in surveillance of drug-resistant trends in a given setting since a high proportion of drug-resistant cases in young age groups could be indicative of recent transmission as opposed to that in older age groups. High proportions of drug resistance in older age groups could be an indicator of the reactivation of old infections.

Over one billion people are reported to be smokers worldwide, 80% of them reside in low- and middle-income countries, many of which also have a high TB burden [29]. In this study, active smoking was not significantly associated with RR-TB but many studies have shown that smoking is significantly associated with increased risks of tuberculous infection [30], morbidity, treatment outcomes [31], and drug resistance [3]. The role that cigarette smoke plays in the pathogenesis of tuberculosis is related to ciliary dysfunction, to a reduced immune response, and defects in the immune response of macrophages, with or without a decrease in the CD4 count, increasing susceptibility to infection with *Mycobacterium tuberculosis* [32], though we did not find any link between cigarette smoking and drug resistance, we think an increase primary and secondary TB transmission, TB relapse, and poor treatment compliance would eventually result in drug resistance. This situation can however be avoided by improving compliance to TB treatment and the application of measures to promote the cessation of smoking.

According to WHO [33], poverty is a strong predictor of TB. New TB infection is not just the product of poverty, but also creates poverty. Understanding the connection between TB and poverty is an important first step towards breaking this vicious cycle. Fighting TB and poverty together is necessary to accelerate economic and social growth and consequently reduce the global burden of TB. In this study, though there was no significant association between monthly income below 50.000 FCFA and TB infection. Persons with incomes of less than 50.000 FCFA predominantly developed RR-TB. Poverty facilitates the transmission of *Mycobacterium tuberculosis*, primarily through (1) its influence on living conditions, such as people living in overcrowded and poorly ventilated homes, (2) prolonged diagnostic delay, and (3) increased vulnerability due to malnutrition and/or HIV infection. Some studies have equally argued that beyond a certain threshold, the exact level of poverty has little influence on transmission risk since living conditions remain conducive. Such studies [34] lay more importance on living conditions rather than the exact level of poverty.

This study reveals that incarceration was statistically associated with RR-TB development. This finding was in line with other studies [35, 36] which show that incarceration was a risk factor for developing TB and subsequent drug resistance. WHO [37] reports show that the level of TB in some prisons is 100 times higher than that of the civilian population and may account for up to 25% of a country's burden of TB with 24% of these cases developing MDR-TB. Factors such as poverty, late diagnosis, inadequate treatment, overcrowding, poor ventilation, and repeated prison transfers may encourage the transmission of TB infection and subsequence drug resistance.

Multivariate analysis of all the factors demonstrates only previous contact with TB patient and year of enrolment that were independent associated with RR-TB. This indicates that before the introduction of the Gene Xpert MTB/RIF and Line Probe assays, drug resistance rates were high more than 3 times in 2016 compared to 2017.

## 5. Conclusion

In univariate analysis, this study shows that age greater than 60 years, being married, and incarcerated are predictors of drug resistant-TB, while the year of patient enrolment and previous exposure to TB treatment were independent predictors of drug resistance in the Littoral region of Cameroon. Though this work was limited to molecular techniques for drug resistance detection and could result in underreporting of drug resistance strains, it remains valid because it highlights the presence of rifampicin resistant-TB, multidrug-resistant TB, and extensive drug-resistant TB cases.

## Figures and Tables

**Table 1:** Clinical scoring system for presumptive TB cases.

Clinical presentation	Score	
	Yes	No
Age ≤ 59	1	0
Cough > 2 weeks	2	0
Hemoptysis	2	0
Weight loss	2	0
Fever	1	0
Night sweats	3	0
History past TB infection	2	0
Total score	13	0

**Table 2:** Sociodemographic characteristics of 1665 *Mycobacterium tuberculosis* infected participants in the littoral region of Cameroon.

Characteristics	Categories	Frequency	Percentage (%)
Gender	Female	612	36.8
	Male	1053	63.2
Age groups (years)	<16	94	5.6
	16-30	566	34. 0
	31-45	640	38.4
	46-60	283	17.0
	> 60	82	4.9
Marital status	Married	924	55.5
	Single	741	44.5
Current incarceration	Yes	297	17.8
	No	1368	82.2
Income level (XAF)	< 50 000 (~100 USD)	1376	82.6
	50,000-100,000	120	7.2
	> 100,000	169	10.2
Patient category	New cases	435	26.1
	Defaulters	70	4.2
	Failure	63	3.8
	Relapse	1097	65.9
Location of TB	Extrapulmonary TB	60	3.6
	Pulmonary TB	1605	96.4
HIV-1 status	Negative	1225	73.6
	Positive	440	26.4

**Table 3:** Indicators of drug resistance.

Characteristics	Resistant *n* (%)	Sensitive *n* (%)	Crude odds ratio (95% CI)	*p* value	Adjusted odds ratio (95% CI)	*p* value
Gender						
Female	37 (4.6)	776 (95.4)	1		1	
Male	49 (5.8)	803 (94.2)	1.28 (0.83–2.0)	0.27	1.18 (0.73-1.93	0.50
Age group (years)						
< 16	2 (2.1)	92 (97.9)	1		1	
16-30	28 (4.9)	538 (95.1)	2.08 (0.91-4.72)	0.081	1.87 (0.75-4.65)	0.177
31-45	30 (4.7)	610 (95.3)	2.20 (0.97-4.98)	0.059	2.05 (0.85-4.95)	0.110
46-60	18 (6.4)	265 (93.6)	1.59 (0.67-3.80)	0.296	1.30 (0.52-3.23)	0.575
>60	8 (9.8)	74 (90.2)	4.98 (1.02-24.39)	0.047	2.67 (0.51-14.1)	0.244
Marital status						
Single	26 (3.5)	715 (96.5)	1		1	
Married	60 (6.5)	864 (93.5)	1.91 (1.19-3.06)	0.006	1.51 (0.89-2.55)	0.128
Year of enrolment						
2017	49 (3.7)	1288 (96.3)	1		1	
2016	37 (11.3)	291 (88.7)	3.34 (2.14-5.22)	< 0.001	3.21 (2.01-5.11)	< 0.001
Incarceration						
No	63 (4.6)	1305 (95.4)	1			
Yes	23 (7.7)	274 (92.3)	1.74 (1.06-2.85)	0.027	1.55 (0.83-2.92)	0.169
Income level (XAF; 50,000 XAF = ~100 USD)						
> 100,000	10 (5.9)	159 (94.1)	1		1	
50,000-100,000	8 (6.7)	112 (93.3)	1.14 (0.44-2.97)	0.795	1.48 (0.52-4.21)	0.462
< 50,000	68 (4.9)	1308 (95.1)	0.83 (0.42-1.64)	0.585	1.03 (0.50-2.14)	0.938
Previous TB history						
No	19 (4.4)	416 (95.6)	1		1	
Yes	67 (5.4)	1163 (94.6)	1.26 (0.75-2.12)	0.382	1.10 (0.59-2.04)	0.776
HIV-1 status						
Negative	58 (4.7)	1167 (95.3)	1		1	
Positive	28 (6.4)	412 (93.6)	1.37 (0.86-2.18)	0.185	1.39 (0.86-2.25)	0.179
Previous contacts with a TB case						
No	58 (4.4)	1256 (95.6)	1		1	
Yes	28 (8.0)	323 (92.0)	1.88 (1.18–3.0)	0.007	1.90 (1.17-3.10)	0.010
Smoking						
No	71 (5.5)	1209 (94.5)	1		1	
Yes	15 (3.9)	370 (96.1)	0.69 (0.39-1.22)	0.199	0.59 (0.32-1.09)	0.093
Alcohol						
No	35 (4.4)	760 (95.6)	1		1	
Yes	51 (5.9)	819 (94.1)	1.35 (0.87-2.10)	0.179	1.20 (0.74-1.94)	0.470

## Data Availability

The datasets used and/or analyzed during the current study are available from the corresponding author on reasonable request.
